# The *Xa7* resistance gene guards the rice susceptibility gene *SWEET14* against exploitation by the bacterial blight pathogen

**DOI:** 10.1016/j.xplc.2021.100164

**Published:** 2021-01-19

**Authors:** Dangping Luo, Jose C. Huguet-Tapia, R. Taylor Raborn, Frank F. White, Volker P. Brendel, Bing Yang

**Affiliations:** 1 Division of Plant Sciences, Bond Life Sciences Center, University of Missouri, Columbia, MO 65211, USA; 2 Department of Plant Pathology, University of Florida, Gainesville, FL 32611, USA; 3 Department of Biology, Department of Computer Science, Indiana University, Bloomington, IN 47405, USA; 4 Donald Danforth Plant Science Center, St. Louis, MO 63132, USA; 5 Current address: Biodesign Institute Center for Mechanisms of Evolution, Arizona State University, Tempe, AZ 85281, USA

**Keywords:** *Xa7*, TAL effector, *Xanthomonas*, bacterial blight, disease resistance, *SWEET14*

## Abstract

Many plant disease resistance (*R*) genes function specifically in reaction to the presence of cognate effectors from a pathogen. *Xanthomonas oryzae* pathovar *oryzae* (*Xoo*) uses transcription activator-like effectors (TALes) to target specific rice genes for expression, thereby promoting host susceptibility to bacterial blight. Here, we report the molecular characterization of *Xa7*, the cognate *R* gene to the TALes AvrXa7 and PthXo3, which target the rice major susceptibility gene *SWEET14*. *Xa7* was mapped to a unique 74-kb region. Gene expression analysis of the region revealed a candidate gene that contained a putative AvrXa7 effector binding element (EBE) in its promoter and encoded a 113-amino-acid peptide of unknown function. Genome editing at the *Xa7* locus rendered the plants susceptible to *avrXa7*-carrying *Xoo* strains. Both AvrXa7 and PthXo3 activated a GUS reporter gene fused with the EBE-containing *Xa7* promoter in *Nicotiana benthamiana*. The EBE of *Xa7* is a close mimic of the EBE of *SWEET14* for TALe-induced disease susceptibility. Ectopic expression of *Xa7* triggers cell death in *N. benthamiana*. *Xa7* is prevalent in *indica* rice accessions from 3000 rice genomes. *Xa7* appears to be an adaptation that protects against pathogen exploitation of *SWEET14* and disease susceptibility.

## Teaser

Bacterial TAL effectors hijack host gene expression to mediate disease susceptibility, and host plants counteract TAL effectors with resistance genes to thwart pathogen infection. This study reports the cloning of *Xa7*, demonstrates its induction by the essential virulence TAL effectors AvrXa7 and PthXo3, and documents its prevalence in *indica* rice cultivars.

## Introduction

Crop plants suffer detrimental effects from plant diseases and pests, which cause global yield losses of about 20% each year (Oerke 2006; Savary et al., 2019). To counteract disease, host plants have evolved innate immunity mechanisms that work against pathogens mainly through a diverse array of plant genes and gene products that recognize molecular signals from the pathogens (Spoel and Dong 2012). Conceptually, resistance is mediated by two general pathways. In the first pathway, membrane-bound receptors recognize conserved small molecules, often of pathogen origin, the so-called pathogen-associated molecular patterns, and trigger basal and broad immunity against the invading pathogens (Jones and Dangl 2006). Many host- and cultivar-specific pathogenic fungi and proteobacteria have evolved processes that suppress basal immunity (Cook et al., 2015; Monteiro and Nishimura 2018). Plants, in turn, have evolved a second layer of defense, the so-called effector triggered immunity (ETI), which involves the specific recognition of immunity-suppressive effectors (Jones and Dangl 2006). Major resistance (*R*) genes are adaptive components of the plant defense system that arise from selective pressure exerted by virulent pathogen populations. In some cases, pathogens can overcome ETI through mutation or loss of *R* gene-specific effector genes or the acquisition of new effectors that, in turn, suppress ETI (Jackson et al., 1999; Feng and Zhou 2012).

Bacterial blight (BB) of rice, caused by the γ-proteobacterium *Xanthomonas oryzae* pathovar *oryzae* (*Xoo*), is among the most damaging diseases in a wide range of South Asian rice-producing areas and also poses a threat in some African countries (Niño-Liu et al., 2006). *R* gene deployment is the most economically sound and environmentally friendly means to control BB, and many BB-specific *R* genes have been identified and characterized at the molecular level (Song et al., 1995; Yoshimura et al., 1998; Yang et al., 2000; Iyer and McCouch 2004; Sun et al., 2004; Gu et al., 2005; Chu et al., 2006; Xiang et al., 2006; Liu et al., 2011; Tian et al., 2014; Wang et al., 2015; Hu et al., 2017; Ji et al., 2020; Zhang et al., 2020). The cognate effectors that recognize *R* genes, as in other proteobacterial disease complexes, are commonly effectors of the type III secretion pathway. Historically, type III effectors with a cognate *R* gene are named Avr effectors (Leach and White 1996). All the known cognate type III Avr effector/*R* gene pairs in BB involve a subset of type III effectors known as transcription activator-like effectors (TALes).

TALes of *Xanthomonas* represent the largest subgroup of type III effector proteins in plant pathogenic bacteria. For the most part, they function as transcription factors that promote the expression of host genes by binding to sequence-specific promoter segments, referred to here as effector binding elements (EBEs). Consequentially, expression of the host susceptibility (*S*) gene enhances the disease process. TALes of *Xoo* that have a dramatic effect on virulence and host susceptibility are referred to as major TALes and are known to target three members of the sucrose transporter, or SWEET, gene family. In the absence of SWEET gene expression, *Xoo* strains are virtually nonpathogenic, and every *Xoo* strain examined to date has at least one gene for a major TALe (Oliva et al., 2019). Rice cultivars have adapted to TALe-mediated virulence by the acquisition of a genetically dominant *R* gene class defined by TALe-specific expression that triggers a state of resistance. Rice *R* genes with TALe-specific expression include *Xa27*, *Xa10*, and *Xa23* (Gu et al., 2005; Tian et al., 2014; Wang et al., 2015). TALe-mediated *R* gene expression has also been demonstrated for *Bs3* and *Bs4C-R* in pepper (*Capsicum* sp*.*) (Römer et al., 2009; Strauss et al., 2012).

*Xa7* is a dominant *R* gene of rice that confers resistance to *Xoo* strains that harbor the cognate major TALe AvrXa7 (Hopkins et al., 1992). The AvrXa7 effector has a dual function: as a virulence factor, it induces the rice *S* gene *SWEET14*, which encodes a sucrose efflux transporter, and as an avirulence factor, it also triggers *Xa7*-mediated resistance. AvrXa7 targets an overlapping EBE of the *S* gene *SWEET14* with a second major TALe, PthXo3. Although the identity and mechanism of *Xa7* are unknown, it has been shown to confer resistance to all six Japanese *Xoo* races or sub-races and 4 of 10 Philippine *Xoo* races (Ogawa et al., 1991). *Xoo* races are defined by the set of *R* genes in a given group of rice cultivars with which the strains are incompatible. The broad spectrum of *Xa7* makes it an important *R* gene in rice breeding programs (Ogawa et al., 1991; Hsu et al., 2020). The pathogen gene *avrXa7* was found in 11 of 33 fully sequenced Asian *Xoo* strains, whereas *pthXo3* was found in 12 of the 33 strains. No strain contained both *avrXa7* and *pthXo3* (Oliva et al., 2019). *Xa7* has been found to retain effectiveness under field conditions (Bai et al., 2000) and to perform better at high temperatures (Webb et al., 2010; Dossa et al., 2020), which are reported to compromise the function of some *R* genes. Efforts to map *Xa7* have placed the gene on chromosome 6 (Kaji and Ogawa 1995; Porter et al., 2003; Chen et al., 2008; Zhang et al., 2009). In this study, we present evidence for the identity of *Xa7* based on fine mapping, gene expression assays, and CRISPR-mediated gene editing.

## Results

### Fine mapping of *Xa7* from IRBB7

**Figure 1:**
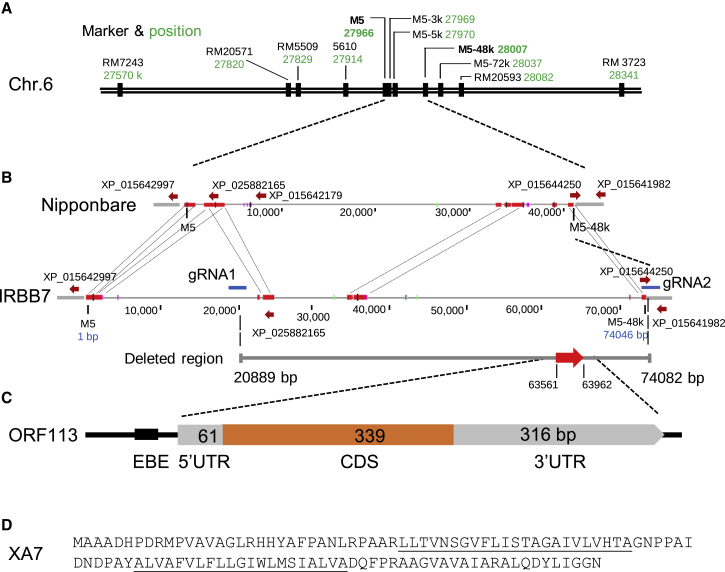
Fine mapping of *Xa7*. **(A)** Physical map of *Xa7* locus and associated markers on chromosome 6. The individual molecular markers are indicated as the genomic coordinates (in kilobases and in green) on chromosome 6 of the Nipponbare reference genome. **(B)** Genomic regions delimited by two markers (M5 and M5-48k) in Nipponbare and IRBB7. Annotated genes in Nipponbare are shown with red arrows, and those in IRBB7 are shown in blue. Red bars linking Nipponbare and IRBB7 indicate syntenic regions. Two Cas9 guide RNAs for the deletion of a 53-kb region within the *Xa7* candidate gene in IRBB7 are denoted by red arrows. **(C)** Schematic graph of *Xa7*. The gene structure is depicted as EBE for AvrXa7, 5′ UTR for the 5′ untranslated region, and 3′ UTR for the 3′ untranslated region. CDS, coding sequence. **(D)** XA7 amino acids. Single letters of amino acids are used. The transmembrane sequences are underlined.

To fine map *Xa7* in IRBB7 (an *indica* rice variety carrying *Xa7*), the first mapping population was created by crossing the near-isogenic line IRBB7 with the recurrent parental cultivar IR24 (Ogawa et al., 1991). For mapping, 220 F2 plants were phenotyped by inoculation with the *avrXa7-carrying Xoo* strain PXO86*,*and genotyped using the previously reported *Xa7*-linked marker M5 (Porter et al., 2003). A set of 10 newly developed molecular markers linked to M5 were also identified and used to further genotype the plants (see Supplemental Table 1 for markers and Supplemental Table 2 for oligonucleotides). *Xa7* was mapped to an interval defined by the markers RM7243 (three recombinants) and RM20593 (two recombinants) and was shown to co-segregate with the markers M5, M5-5k, and M5-48k (Figure 1A). These results indicate that *Xa7* is located within a region of 512 kilobases (kb) between RM7243 and RM20593 relative to the reference genome of the cultivar Nipponbare. An additional 17 000 members from an F2 population of IRBB7 and Nipponbare were screened for recombinants between RM7243 and RM20593, and the recombinants were phenotyped for genetic association analysis. Based on a number of polymorphic markers (Supplemental Table 1), *Xa7* was shown to reside within a region corresponding to the 41.3-kb region between M5 and M5-48k on the Nipponbare reference genome (Figure 1B).

Short and long sequencing reads obtained from IRBB7 DNA by Illumina and Nanopore sequencing were used for *de novo* assembly of the *Xa7* region across markers M5 and M5-48k, resulting in a genomic sequence of 74 kb. PCR amplification and sequencing of the amplicons were also performed to validate the accuracy of the sequencing data, and the sequence was aligned with the related region from Nipponbare (Figure 1B). The regions are syntenic and include homologous genes that encode the IRGSP GenBank protein accessions XP_025882165.1 (only the 402 amino acid [aa] C-terminal exon is conserved in IRBB7), XP_015642179.1 (not present in IRBB7), XP_015644250.1 (perfectly conserved), and XP_015641982.1 (perfectly conserved). All proteins are annotated as “uncharacterized.” XP_015641982.1 contains a common protein–protein interaction motif of about 100 aa, known as the BTB/POZ domain. The IRBB7 contig has a GC content of approximately 45%, and about 62% of the sequence matches transposable elements as determined by RepeatMasker (version open-4.0, http://www.repeatmasker.org) using the RITE database (Copetti et al., 2015).

### Deletion of the *Xa7* region

**Figure 2:**
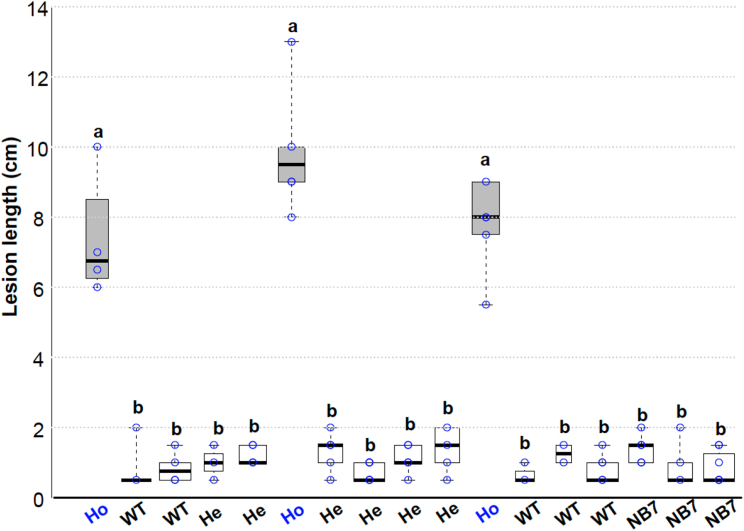
Disease phenotypes of progeny from the NB7 CRISPR T0 line. Individual plants with genotypes homozygous (Ho) and heterozygous (He) for the 53-kb deletion and wild-type (WT) plants were inoculated with PXO86. Lesion lengths were measured 12 days after inoculation on three to five leaves of individual plants. Individual segregants were genotyped for the presence or absence of the 53-kb chromosomal fragment deletion by PCR. Ho, segregants homozygous for the 53-kb deletion; He, segregants heterozygous for the 53-kb deletion; WT, progeny homozygous for the WT genotype; NB7, the parent *Xa7* isogenic line. Center lines show the medians; box limits indicate the 25th and 75th percentiles as determined by R software; whiskers extend to the minimum and maximum values; box width is proportional to the square root of the sample size; and data points are plotted as open circles. *n* = 3 to 8 sample points. Treatments with the same lowercase letter are not significantly different at *p* < 0.05.

To further confirm the location of *Xa7*, a line named NB7, which was derived from an IRBB7 and Nipponbare cross and is resistant to PXO86, was used to delete 53 kb of the IRBB7 *Xa7* region using CRISPR-Cas9 with two guide RNAs (Figure 1B, gRNA1 and gRNA2). NB7 is transformable due to its Nipponbare genetic background. PCR with deletion-specific and internal primers showed that one transgenic line, nb7-1, contained a large 53-kb deletion delimited by the two guide RNAs in one of its chromosomal copies (Supplemental Figure 1; see Supplemental Table 2 for oligonucleotide information). The nb7-1 line was resistant to disease after inoculation with *Xoo* strain PXO86, indicating that it retained a copy of *Xa7* and was heterozygous for the deletion. Susceptibility was shown to co-segregate with the homozygous deletion in the T1 population (*n* = 5/24) using primers that could detect the wild-type and deleted regions (Figure 2).

### Gene expression from the *Xa7* region

We hypothesized that *Xa7* could be distinguished from the other annotated genes in the 53-kb region based on its TALe-mediated expression. RNA samples from IRBB7 infected with PXO86 and the *avrXa7* mutant MX53 were subjected to RAMPAGE analysis, which combines RNA annotation and mapping of the respective promoters (Batut and Gingeras 2013; Raborn and Brendel 2019). The transcription start site (TSS)-adjacent sequences of transcribed genes in the unique 53-kb region were captured, and the RAMPAGE reads were used to project the transcript abundance of individual genes in the two treatments (with and without *avrXa7*). We used the Bioconductor TSRchitect package (Raborn et al., 2017) to identify transcription start regions (TSRs) in the contig, and the edgeR package (Robinson et al., 2010; McCarthy et al., 2012) to assess differential expression. Only one strongly induced TSR was found in the 53-kb region, in positions 63 494–63 513 (predominant TSS at 63 503; Figure 1), and it showed a 147-fold increase in expression after treatment with PXO86 relative to MX53 (Supplemental Figure 2). We refer to this transcript as *R-Xa7* (Figure 1B, red arrow).

Sixty-eight base pairs downstream of the predominant *R-Xa7* TSS site is an open reading frame (ORF) of 342 bp (including the stop codon) designated ORF113. A 726-bp cDNA, encompassing ORF113, a 68-bp 5′ UTR, and a 316-bp 3′ UTR, was isolated by screening a cDNA library derived from IRBB7 infected with PXO86 (Figure 1C, Supplemental Information 1). ORF113 was predicted to encode a small protein of 113 aa that showed no significant similarity to known R proteins in the database (Figure 1D). The gene from IRBB7 is hereafter tentatively referred to as *Xa7*.

**Figure 3:**
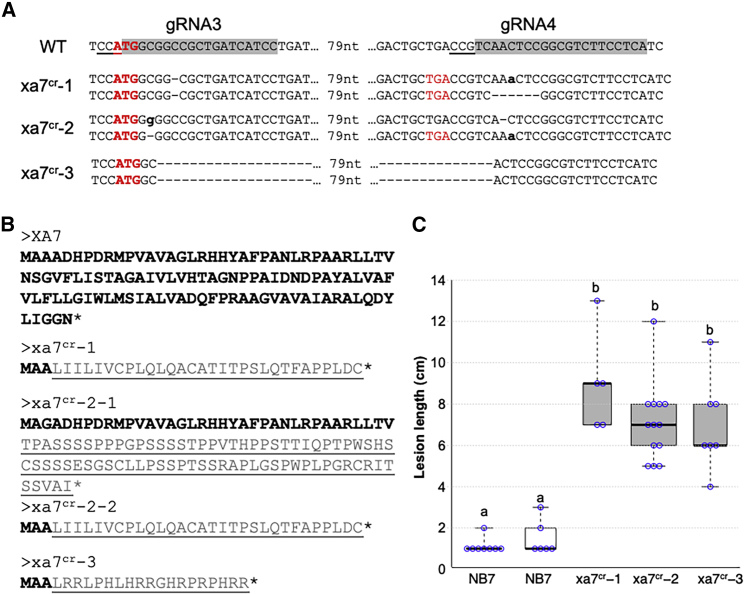
Disease phenotypes of *Xa7* mutant lines. **(A)** Genotypes of three CRISPR lines with deletions and insertions at two guide RNA target sites. Guide RNA target sequences are shaded, and the adjacent Cas9 PAM sequences are underlined. Start codons are in red and bold, and stop codons are in red. Inserted nucleotides are in bold lowercase letters, and dashed lines indicate deleted nucleotides. **(B)** Genotypes of three CRISPR lines with *Xa7* knocked out. Predicted amino acids encoded by different alleles are shown, with WT sequences in bold and new amino acids due to frameshift mutations underlined. **(C)** The isogenic line NB7 and three CRISPR lines containing *Xa7* knockout mutations were inoculated with PXO86. Lesion lengths were measured as for Figure 2. Treatments with the same lowercase letter are not significantly different at *p < 0.05.*

To corroborate its involvement in *Xa7*-mediated resistance, two sites in *Xa7* were targeted by CRISPR in NB7. Two CRISPR target sites in *Xa7* were chosen to construct two guide RNAs for the transformation of NB7 (Figure 3A, gRNA3 and gRNA4). Sequence analysis of 15 T0 transgenic plants revealed three independent T0 plants in which both alleles were knocked out: xa7^cr^-1, xa7^cr^-2, and xa7^cr^-3. The mutations (1-bp deletion) at the first guide RNA target in xa7^cr^-1 led to a premature stop codon; the mutations (79-bp deletion) in xa7^cr^-2 also led to a premature stop codon in *Xa7*; and the two alleles in xa7^cr^-3 also led to two null mutations in *Xa7* (Figure 3A and 3B). All three altered lines were susceptible to PXO86, indicating that *Xa7*-mediated resistance to PXO86 requires functional *Xa7* (Figure 3C).

**Figure 4:**
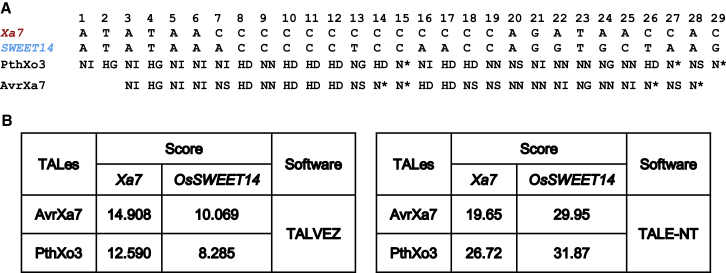
AvrXa7 and PthXo3 are predicted to target two overlapping EBEs in *Xa7*. **(A)** Individual RVDs of the AvrXa7 and PthXo3 central repeats match single nucleotides of the predicted EBE regions in the *Xa7* promoter. Single letters are used for amino acids at the 12th and 13th positions of individual repeats. ∗ The missing amino acid at the 13th position of a particular repeat. The EBE regions for AvrXa7 and PthXo3 in the *S* gene *SWEET14* are shown for comparison. **(B)** The scores of matches between the DNA sequences of putative *Xa7* and *SWEET14* (as controls) and the repeats of AvrXa7 and PthXo3 as predicted by two programs (TALE-NT and TALVEZ). A lower score indicates a higher binding affinity between the RVDs and the target sequence.

To identify the putative EBE for AvrXa7 in *Xa7* (designated *EBE*_AvrXA7_), the sequence upstream of the *Xa7* cDNA was analyzed by the EBE prediction programs TALVEZ and TALE-NT (Doyle et al., 2012; Booher and Bogdanove 2014). Both programs predicted consensus sequences of 26 nucleotides for AvrXa7 and 29 nucleotides for PthXo3 located 134–109 and 136–107 bp upstream of the *Xa7* ATG that exhibited the DNA binding specificity predicted for the repeat regions of AvrXa7 and PthXo3, respectively (Figure 4A). The EBEs of *Xa7* for AvrXa7 and PthXo3 are similar to the corresponding EBEs of *SWEET14* (Figure 4A), and their predicted binding scores are comparable to those of the previously characterized overlapping *SWEET14* EBEs for AvrXa7 and PthXo3 (Figure 4B) (Antony et al., 2010).

### AvrXa7 and PthXo3 induce *Xa7* expression in an *EBE*_AvrXa7_-dependent manner

**Figure 5:**
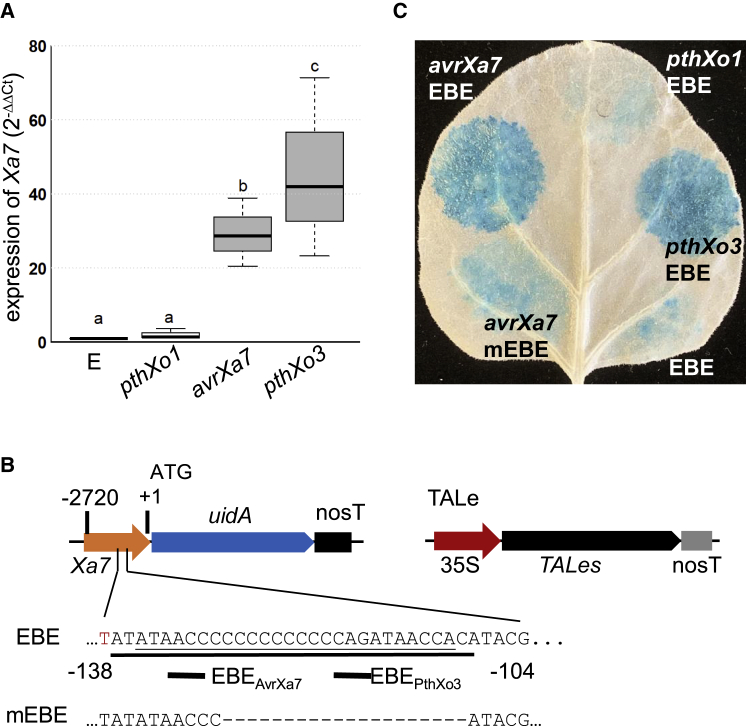
Transient assay of interactions between TALes and a reporter gene fused with the promoter elements of *Xa7*. **(A)** Induction of the *Xa7* candidate gene in IRBB7 by ME2 carrying different TALe genes as revealed by qRT-PCR. **(B)** Constructs of the β-glucuronidase (GUS) gene fused with a promoter fragment (EBE) or a mutated element (mEBE) and of TALe genes under the 35S promoter. **(C)** GUS staining *of N. benthamiana* leaf infiltrated with *Agrobacterium* strains containing different constructs from **(A)**. The leaf was stained with X-Gluc for 1 h and destained with ethanol for 3 days.

To examine the expression of *Xa7* in response to *Xoo* inoculation, qRT-PCR was performed using RNA extracted from inoculated rice leaf tissue and specific primers in *Xa7* after inoculation with *Xoo* strains that varied in major TALe gene content. The strain ME2 is a mutant of PXO99^A^ that has no major TALe gene (Yang and White 2004). Individual major TALe genes were introduced into ME2, and these strains were inoculated on IRBB7. *Xa7* was induced after inoculation with ME2(*avrXa7*) and ME2(*pthXo3*) (Figure 5A). Expression was not observed after inoculation with ME2(*pthXo1*) or ME2, indicating that *Xa7* induction is specific to AvrXa7 and PthXo3 (Figure 5A**)**. TALe-dependent *Xa7* promoter activity was assayed by transient expression using a β-glucuronidase (GUS**)** reporter in *Nicotiana benthamiana*. A 2.7-kb fragment upstream of the ATG of *Xa7* was ligated with the GUS reporter gene (Figure 5B), and the construct (labeled EBE) was co-transferred with CaMV 35S-driven *avrXa7* into *N. benthamiana* leaf cells by agroinfiltration (Figure 5C). Similarly, the reporter construct was co-delivered with *pthXo1* or *pthXo3*. Sites inoculated with *avrXa7* and *pthXo3* displayed high GUS activity (Figure 5C), whereas sites inoculated with the GUS reporter construct (EBE), an empty construct, or 35S-driven *pthXo1* displayed very low GUS activity. When a promoter containing a mutated EBE (labeled mEBE with a 20-bp deletion) was used, GUS activity with *avrXa7* was reduced (Figure 5C).

### Ectopic expression of *Xa7* induces cell death in *N. benthamiana*

**Figure 6:**
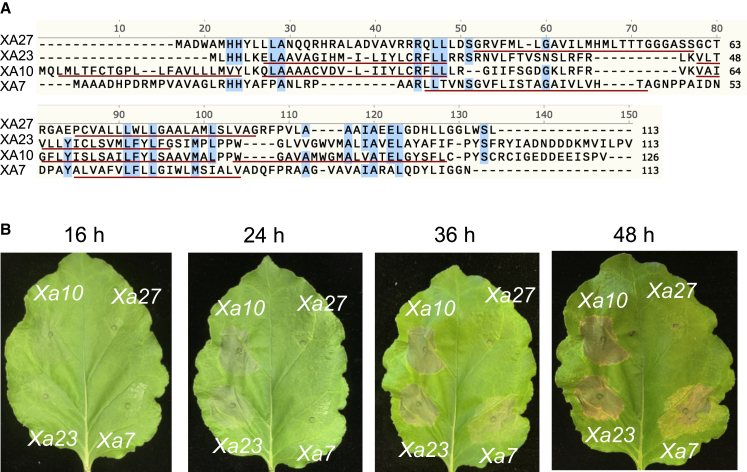
Transient expression of TALe-induced *R* genes in *N. benthamiana*. **(A)** Alignment of predicted amino acids encoded by four executor *R* genes (*Xa7, Xa27, Xa23*, and *Xa10*). Conserved amino acids are shaded in blue. Transmembrane regions are underlined as predicted by TMpred (https://embnet.vital-it.ch/software/TMPRED_form.html). The NCBI COBALT multiple alignment tool was used. Sequences of XA10, XA23, and XA27 are from NCBI accessions AGE45112.1, AIX09985.1, and AYY54165.1, respectively. **(B)** Cell death (rapid localized cell collapse within 48 h) in *N. benthamiana* leaves. The four genes were placed under the control of the 35S promoter and transiently expressed through agroinfiltration. Different time points are indicated above each panel.

Although all the TALe-dependent executor R proteins of rice share some amino acid identities, it is unclear whether the proteins are phylogenetically related, with the exception of XA10 and XA23 (Figure 6A). Ectopic expression of *Xa10* and *Xa23* triggers cell death in *N. benthamiana* (Tian et al., 2014; Wang et al., 2015), but the effect of *Xa27* and *Xa7* is unknown. To investigate whether *Xa7* can function in *N. benthamiana*, the 35S promoter (35S) was placed immediately upstream of the translation start codon of the *Xa7*, *Xa10*, *Xa23*, and *Xa27* ORFs, and each construct was delivered into *N. benthamiana* leaves by agroinfection. A weak HR (hypersensitive response) for *Xa7*, *Xa10*, and *Xa23* was visible at 16 h after infiltration, and HR was pronounced at 48 h after infiltration, although the degree of cell death induced by *Xa7* appeared lower than that induced by *Xa10* and *Xa23* (Figure 6B). No visible HR occurred after the transient expression of *Xa27* (Figure 6B)*.*

### Spectrum of *Xa7* resistance against various *Xoo* strains

**Figure 7:**
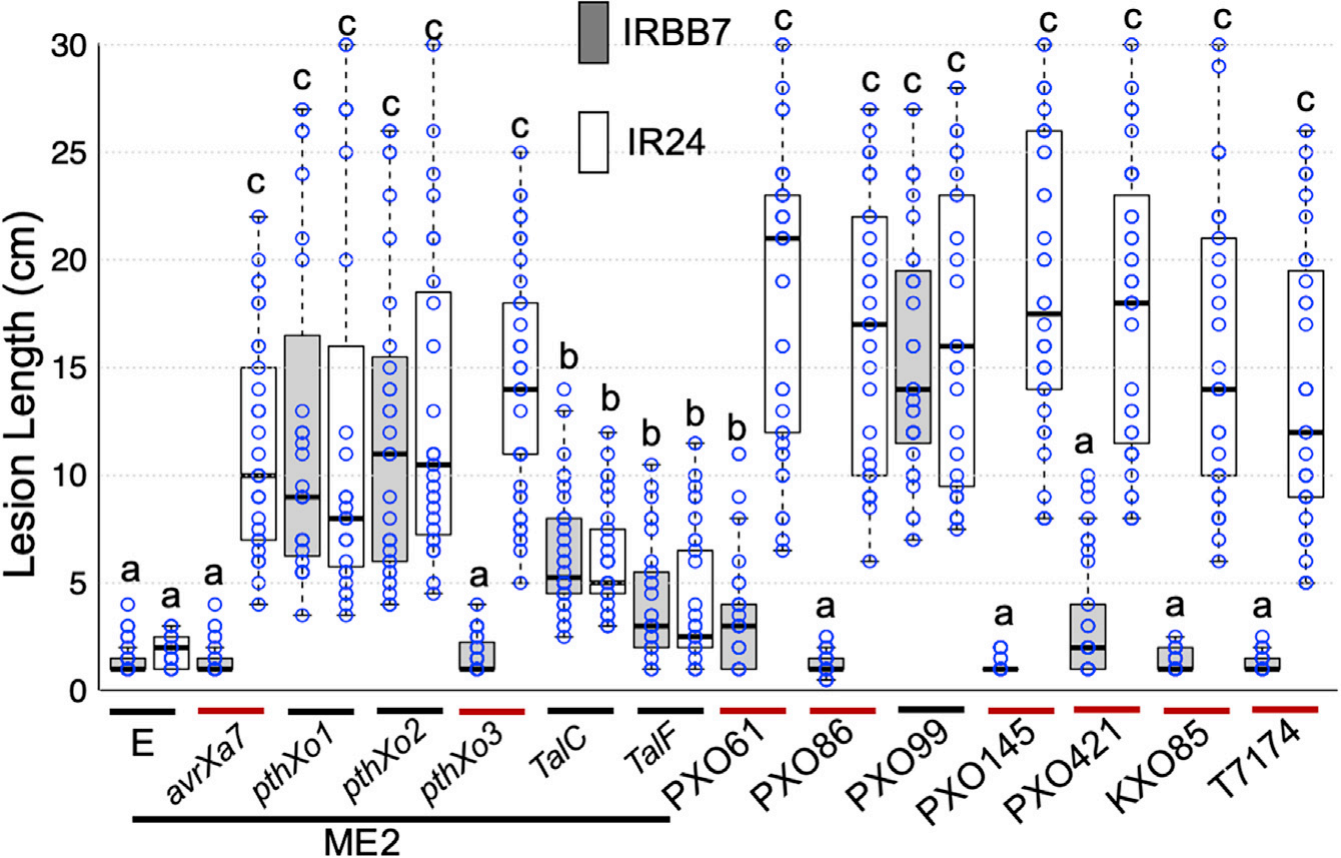
*Xa7* confers resistance to *Xoo* strains containing *avrXa7* or *pthXo3*. Rice plants of IR24 (clear boxes) and IRBB7 (filled boxes) were inoculated with strain ME2 carrying different major TALe genes. Individual strains are identified on the basis of the major TALe gene. Field isolates are specified below individual graphs to the left of the ME2-derived strains. Center lines indicate the median lesion length; box limits indicate the 25th and 75th percentiles as determined by R software; whiskers extend to the minimum and maximum values; box width is proportional to the square root of the sample size; and data points (*n* = 26–42) are plotted as open circles. Treatments with the same lowercase letter are not significantly different at *p* < 0.05.

We next examined the spectrum of *Xa7* resistance against all known major TALe genes and representative *Xoo* strains. Six major TALe genes (*avrXa7*, *pthXo1*, *pthXo2*, *pthXo3*, *TalC*, and *TalF*) that target three cognate SWEET-related *S* genes in rice were transferred into ME2. ME2 is not pathogenic in either IR24 or IRBB7 due to loss of the endogenous copy of *pthXo1* and the resulting inability to induce any SWEET gene. Individual ME2 complementation strains carrying *avrXa7* and *pthXo3* caused susceptibility in IR24 and resistance in IRBB7, whereas the other four TALe genes in ME2 caused susceptibility in both IR24 and IRBB7, indicating that *Xa7* is specific to *avrXa7* and *pthXo3* (Figure 7). Seven representative field isolates of *Xoo* are known to carry *avrXa7* or *pthXo3* or lack either gene (Oliva et al., 2019). Only isolates carrying *avrXa7* or *pthXo3* triggered resistance (Figure 7). ME2(*pthXo3*) resulted in an incompatible interaction, and PXO61, which contains *pthXo3*, was scored as moderately susceptible in comparison to the PXO99 and PXO86 reactions. IRBB7 has been scored as moderately susceptible to resistant to PXO61 in previous tests (Institute 2006).

### Prevalence of *Xa7* locus in other species and rice cultivars

*Xa7* homologs have been identified in other species. Homologs exist in wild rice species (*O. punctata* and *O. longistaminata*), sorghum, *Setaria*, and panicgrasses (*P. hallii* and *D. oligosanthes*). The C termini of XA7 homologs are more conserved than the N termini (Supplemental Figure 3A and 3B). To determine whether representative genes could cause cell death in *N. benthamiana*, the coding sequences of six homologous genes were each constructed under the control of the 35S promoter and expressed ectopically by agroinfiltration of *N. benthamiana* (Supplemental Information 1). Among these constructs, only *Ol_Xa7* resulted in an HR at 48 h. *Op_Xa7* appeared to cause a weak cell death response compared with that of *Xa7* (Supplemental Figure 3C). No obvious cell death phenotype was observed with the remaining homologs (Supplemental Figure 3C).

A total of 294 accessions from a scan of 3000 rice genome sequences contained *Xa7* coding sequences (Supplemental Data 1). A 3171-bp region of the IRBB7 *Xa7* locus, including 842 bp upstream and 2014 bp downstream of the *Xa7* coding sequence, was used as the reference to assemble reads from these 294 accessions, generating 294 contigs that contained *Xa7* and its flanking sequences. Due to the lower sequencing coverage of some accessions and the limitations of next generation sequencing technology to reveal 13 consecutive Cs, only nine contigs contained the *Xa7* EBEs for AvrXa7 and PthXo3. The EBEs could not be unambiguously identified in the majority of contigs (Supplemental Data 1).

Among the 294 accessions, the majority (*n* = 185, 63%) are *indica*, 72 are *japonica* (24%), 22 are Aus/boro, 14 are Basmati/sadri, and seven are intermediate types (Supplemental Data 1). Geographically, India has the most accessions (*n* = 58), followed by China (*n* = 41), Bangladesh (*n* = 34), Indonesia (*n* = 26), the Philippines (*n* = 22), and Cambodia (*n* = 19) (Supplemental Data 1).

## Discussion

*Xa7* has mysteriously evaded cloning endeavors for the past 20 years. Here, multiple approaches provide evidence that our postulated candidate gene is indeed the cognate *R* gene for the TALe AvrXa7. Three critical observations are that (1) the gene lies within the region of markers previously associated with the *Xa7* locus; (2) the deletion of the region and specific mutations in ORF113 eliminate *Xa7*-mediated resistance; and (3) the gene is expressed in an AvrXa7-dependent manner upon infection. That the AvrXa7-mediated expression of *Xa7* is directed by a sequence-specific element in its promoter was corroborated by transient expression assays in *N. benthamiana* leaves. The GUS fusion was not expressed in the absence of AvrXa7 or another TALe (PthXo1) that has an alternate binding specificity. Disruption of the EBE by a 20-bp deletion also interfered with AvrXa7-dependent transient expression. The experiments also revealed that the TALe PthXo3 functions as an allele of AvrXa7 and directs *Xa7*-mediated resistance. The PthXo3-dependent activity of *Xa7* explains its relatively broad activity against extant Asian strains of *Xoo*. The gene for PthXo3 was originally cloned from PXO61, a strain isolated in the Philippines. IRBB7 has been scored variously as moderately susceptible (or resistant) to resistant to PXO61 (Ogawa et al., 1991; Lee et al., 2003; Zhang et al., 2009; Xu et al., 2012). Here, we demonstrate that a variety of strains carrying the endogenous and transferred *pthXo3* are associated with *Xa7*-mediated resistance. Genes for AvrXa7 and PthXo3 are found in many extant strains of *Xoo* (Oliva et al., 2019). Identification of *Xa7* not only advances our understanding of molecular and genetic mechanisms of disease resistance but also facilitates the marker-assisted breeding of *Xa7* into elite rice cultivars for broad resistance.

Cloning of *Xa7* increases the number of executor *R* genes to four (*Xa10, Xa23, Xa27*, and *Xa7*). These genes form a group of unique *R* genes in rice and can be further divided into three subgroups with *Xa10* and *Xa23* in one group and *Xa27* and *Xa7* as single members of two additional groups. *Xa27* has four *Xa27*-like genes (Os06g39810, Os06g07150, Os06g39800, and Os06g39860) in Nipponbare alone (Li et al., 2013). *Xa10/Xa23* has two homologous genes (Os11g37620 and Os11g37570) in the Nipponbare genome (Wang et al., 2017). There is only one *Xa7* homolog (Os11g26900) in the Nipponbare genome, and the two share 57% identity at the amino acid level. No evidence has been found to indicate that the TALe-induced *R* genes serve any purpose other than disease resistance. Conversely, the cognate TALe genes *avrXa10* (for *Xa10*), *avrXa23* (for *Xa23*), and *avrXa27* (*Xa27*) lack a detectable virulence contribution to their respective *Xoo* isolates. AvrXa7 and PthXo3, on the other hand, are major virulence determinants for their respective *Xoo* isolates (Yang et al., 2000; Yang and White 2004). Inactivation of the corresponding genes, *avrXa7* or *pthXo3*, renders these isolates almost nonpathogenic (Hopkins et al., 1992; Yang and White 2004). The alteration of such virulence TALes to evade recognition by cognate *R* genes imposes a physiological and ecological fitness penalty on *Xoo* strains due, in this case, to the loss of elevated *SWEET14* expression. *Xa7* is thus far unique in that its EBE mimics that of the *S* gene, guarding against pathogen exploitation of *SWEET14* in healthy cells by triggering the death of infected cells that are injected with either of the two major TALes.

Several examples show that the transient overexpression of executor *R* genes in *N. benthamiana* can induce cell death, which may mimic the HR phenotype in the host plant/microbe interaction (Romer et al., 2009; Strauss et al., 2012; Tian et al., 2014; Wang et al., 2015; Wang et al., 2017; Wang et al., 2018). In this study, we compared the ability of four executor genes cloned from rice to induce cell death in *N. benthamiana*. The appearance of the HR showed that *Xa7* has a moderate ability to induce cell death relative to *Xa10* and *Xa23*, both of which trigger cell death much more rapidly and strongly. No visible cell death was observed in *N. benthamiana* when *Xa27* was overexpressed (Figure 6B) (Tian et al., 2014). *Xa27* was reported as an *R* gene that triggered a strong HR in response to AvrXa27 in rice (Gu et al., 2005), and *Xa27*-like genes activated by designer TALes that targeted the promoters of *Xa27*-like genes also mediated strong HR in rice (Li et al., 2013). Together with the observation of the diverse abilities of six *Xa7* homologs to induce cell death in *N. benthamiana*, our results therefore show that the mechanism of cell death induction in *N. benthamiana* may differ from the HR triggered in rice by the corresponding avirulence TALe gene.

All four executor *R* genes in rice encode small proteins: XA27, XA23, and XA7 consist of 113 aa, and XA10 consists of 126 aa (Figure 6A). XA27, the first executor *R gene* identified, is predicted to contain two transmembrane domains (Gu et al., 2005). The N terminus of XA27 is also predicted to contain a signal anchor-like sequence, leading to the cellular localization of XA27-green fluorescent protein to the apoplast and wall of xylem cells. Alterations to the hydrophobic nature of the signal anchor-like sequence change the location and resistance activity of XA27 to an avirulent strain of *Xoo* (Wu et al., 2008). On the other hand, XA10 is predicted to contain four transmembrane domains and localizes in the endoplasmic reticulum (ER) (Tian et al., 2014). XA10 was found to be associated with ER Ca^2+^ depletion in plant and HeLa cells. Mutations that render the protein unable to deplete ER Ca^2+^ and to cause cell death in *N. benthamiana* concomitantly abolish *Xa*10-mediated resistance in rice (Tian et al., 2014). Similarly, *Bs4C-R*, induced by AvrBs4 for the resistance of pepper to *Xanthomonas campestris* pathovar *vesicatoria*, encodes a 164-aa protein of unknown function (Strauss et al., 2012). Bs4C-R is predicted to contain four transmembrane motifs, and a fluorescent fusion protein of BS4C-R was shown to localize in the ER membrane in *N. benthamiana*. BS4C-R causes cell death in *N. benthamiana* when ectopically expressed (Wang et al., 2018). Significantly, *Xa10* promoter-Bs4C genes confer rice resistance to *Xoo* strains carrying *avrXa10* (Wang et al., 2018). One exception to the small TALe-induced R proteins is *Bs3* of pepper, which encodes 342 amino acids. Its gene product is homologous to the flavin-dependent monooxygenases (Römer et al., 2007), a group of enzymes that catalyze a wide range of chemo-, regio-, and enantio-selective oxygenation reactions (Huijbers et al., 2014). In this study, XA7 is predicted to possess two transmembrane domains (Figure 6A), but its cellular localization remains to be characterized. Genome editing, especially base editing that induces DNA substitutions (Anzalone et al., 2020), will provide a robust tool with which to dissect the structure-function requirements of XA7 in an endogenous context.

## Methods

### Plant materials, bacterial strains, medium, and growth conditions

The *indica* rice variety IR24, the recurrent near-isogenic line IRBB7 with *R* gene *Xa7*, and the *japonica* variety Nipponbare were kindly provided by the International Rice Research Institute and the U.S. National Small Grains Collection. The rice line NB7 was a segregant from a cross of IRBB7 and Nipponbare that exhibited *Xa7*-mediated resistance activity and the tissue culture trait of Nipponbare. *N. benthamiana* seeds were kindly provided by Dr. Gregory Martin. *Xoo* strains PXO86, PXO99^A^, and PXO99^A^ mutant ME2 and transformants ME2(*avrXa7*), ME2(*pthXo3*), and ME2(*pthXo1*) were from the collection of the Yang laboratory.

All rice plants were grown in the greenhouse and growth chambers with a 12-h 30°C light period and a 12-h 28°C dark period at 60%–75% relative humidity. *Escherichia coli* strains were grown in Luria-Bertani medium supplemented with appropriate antibiotics at 37°C. *Agrobacterium tumefaciens* strains were grown at 30°C. All *Xoo* strains were grown at 28°C on TSA (10 g/l tryptone, 10 g/l sucrose, 1 g/l glutamic acid, 1.5% Difco agar). Antibiotics were used at the following concentrations if required: 100 μg/ml ampicillin, 10 μg/ml cephalexin, 25 μg/ml rifampin, 25 μg/ml kanamycin, and 100 μg/ml spectinomycin.

### Disease assays

The leaf tip-clipping method was used to measure the lesion lengths of blight disease as described previously (Yang and Bogdanove 2013). In brief, an aliquot of the appropriate *Xoo* glycerol stock, stored at −80°C, was streaked onto TSA containing appropriate antibiotics and grown at 28°C for about 3 days. The bacterial cells were harvested from plates, suspended in sterile water, washed twice with water, and resuspended in water; the solution was adjusted to an optical density of 0.5 at 600 nm. Scissor blades were immersed in the *Xoo* suspension and used to clip the tip of a fully expanded leaf. The lesion lengths were measured at 14 days or at the specified days after inoculation. Three replicates with multiple leaves per replicate were examined for each *Xoo* strain. Data were plotted using BoxPlotR (http://shiny.chemgrid.org/boxplotr/). One-way analysis of variance was performed on all measurements. The Tukey honestly significant difference test was used for post-ANOVA pair-wise tests for significance (*p*<0.05).

### CRISPR-Cas9-based gene editing in rice

The CRISPR-Cas9 system used to generate a large chromosomal deletion in the *Xa7* locus and mutations within the *Xa7* coding region was described previously (Zhou et al., 2014). Two guide RNA genes (gRNA1 and gRNA2) targeting two sites spanning the 53-kb *Xa7* locus were constructed in the intermediate guide RNA vector pgRNA-1. The guide RNA gene cassette was mobilized into the Cas9 destination vector pBY02-Cas9-GW through the Gateway reaction using LR clonase (Thermo Fisher Scientific), resulting in pCas9-gRNA1+2. Similarly, two guide RNA genes targeting two sites (gRNA-3 and gRNA-4) in the *Xa7* coding region were combined into pBY02-Cas9-GW, resulting in pCas9-gRNA3+4. Both constructs were transferred into the *Xa7* isogenic line NB7 through the biolistic particle bombardment DNA delivery method. Rice tissue culture and regeneration were performed using methods described previously (Hiei et al., 1994). Genotyping the CRISPR plants from the T0 and T1 generations was performed by PCR amplification of relevant regions and Sanger sequencing of the amplicons.

### Gene expression assays

For RAMPAGE experiments, young leaves of IRBB7 were inoculated with PXO86 and the *avrXa7* knockout mutant MX53 (Hopkins et al., 1992). Total RNA was extracted using the TRIzol reagent (Thermo Fisher Scientific) 24 h after inoculation. Three replicates for each *Xoo* strain were used to construct RAMPAGE libraries for paired-end sequencing as described previously (Batut and Gingeras 2013; Raborn and Brendel 2019). In brief, total RNA was subjected to DNase I treatment, ribosomal RNA depletion, reverse complementary DNA synthesis with custom RAMPAGE-specific oligos, cap-trapping of the 5′-complete cDNA and RNA double-stranded DNA/RNA, streptavidin-based pull-down of the biotinylated DNA/RNA, PCR amplification and size selection of double-stranded DNA, and Illumina-based paired-end sequencing. Library quality was assessed using the Agilent 2200 TapeStation instrument (Agilent Technologies, Santa Clara, CA, USA) at the Indiana University Center for Genomics and Bioinformatics. All computational analyses are documented for reproducibility at https://github.com/BrendelGroup/AllRice following the guidelines proposed in Brendel (2018).

### Transient TALe-specific *Xa7* promoter activity in *N. benthamiana*

*Xa7* promoter fusions to GUS reporter constructs were made using the 2.7-kb promoter region upstream of the *Xa7* ATG after amplification with the oligos Pro2.7kHind-F and Xa7ATG-R from IRBB7 genomic DNA. The amplicon was cloned into pCAMBIA1305 at *Hind*III and *Nco*I through Gibson cloning (Gibson et al., 2009). To construct the *Xa7* promoter-GUS reporter with a mutant AvrXa7 binding element, two fragments of the promoter were amplified with Pro2.7kHind-F and DelEBE-R3 and DelEBE-F3 and Xa7ATG-R from IRBB7 genomic DNA and inserted into pCAMBIA1305 at *Hind*III and *Nco*I. The constructs were transferred into the *Agrobacterium* strain EHA105. The TALe expression constructs were made by cloning the coding regions of *avrXa7*, *pthXo3*, and *pthXo1* under the 35S promoter in pCAMBIA1300 at *Bam*HI and *Spe*I sites. *N. benthamiana* plants were grown under 12 h of light and 12 h of darkness at 25°C and approximately 40%–60% relative humidity. Leaves of 4-week-old *N. benthamiana* plants were used for infiltration with a 1-ml needleless syringe. *Agrobacterium* strain EHA105 that harbored the construct of interest was cultured in Luria-Bertani medium containing 25 mg/l rifampin, 25 mg/l kanamycin, and 100 μM acetosyringone. The bacterial cells were collected through centrifugation and resuspended in Murashige and Skoog medium containing 100 μM acetosyringone, pH 5.8. The cell suspension was adjusted to an OD_600_ of 0.2 for infiltration. For co-inoculation, cells of two *Agrobacterium* strains were mixed in equal volume before infiltration into *N. benthamiana* leaves.

### Sequencing and annotation of the *Xa7* region

Genomic DNA of IRBB7 was extracted using the CTAB method (Porebski et al., 1997). Sequencing was conducted using long-read Oxford Nanopore and Illumina technologies. The assembler Flye (Kolmogorov et al., 2019) was used to create a *de novo* assembly of the IRBB7 genome. The resulting contigs were first corrected by re-mapping Nanopore reads and correcting with the medaka tool. A second correction was performed by mapping highly accurate Illumina reads using Bowtie 2 (Langmead and Salzberg 2012) and the Pilon correction tool (Walker et al., 2014). Gene structure annotation was based on spliced alignment of homologous proteins and transcripts using GenomeThreader (Gremme et al., 2005).

### Survey of the *Xa7* locus from 3000 rice genomes

The complete 3000 rice genome project (3K RGP) database was downloaded from http://gigadb.org/dataset/200001. The database contains ∼11 Tb of raw paired-end Illumina reads from 3010 diverse cultivated rice (*Oryza sativa* L.) accessions. Reads were mapped against the 4-kb region spanning the *Xa7* locus using Bowtie 2 (Langmead and Salzberg 2012). The mapped reads were retrieved from 294 rice lines (including IRBB7), compressed as bam files, and sorted. A consensus fasta file of the *Xa7* region from each rice line was created using SAMtools and BCFtools (Li et al., 2009; Li 2011). The process was performed using a customized Unix pipeline (Supplemental Information 2).

## Funding

This work was partially supported by the United States Department of Agriculture National Institute of Agriculture and Food (2017-67013-26521 to B.Y.), the 10.13039/100000001National Science Foundation (1238189 to F.F.W., V.P.B., and B.Y.; 1741090 to F.F.W.), and subawards to 10.13039/100007165University of Missouri and University of Florida from the 10.13039/501100003484Heinrich Heine University Düsseldorf funded by the 10.13039/100000865Bill & Melinda Gates Foundation [OPP1155704] (B.Y. and F.F.W.).

## Author contributions

B.Y. and D.L. conceived the experiments. D.L. performed the experiments. J.C.H.-T. and F.F.W. conducted Nanopore sequencing of IRBB7, performed genome assembly, and analyzed the 3000 rice genomes for *Xa7* prevalence. R.T.R. constructed and sequenced the RAMPAGE and RNA sequencing libraries. V.P.B. analyzed the RAMPAGE and RNA sequencing data. D.L. and B.Y. wrote the manuscript, and F.F.W. edited the manuscript with input from all other co-authors.

## Accession numbers

The NCBI GenBank accession numbers are MW467883 for the *Xa7* cDNA, MW561276 for the *Xa7* contig, and GSE165583 for the RAMPAGE data. All computational analyses are documented for reproducibility at https://github.com/BrendelGroup/AllRice.

## Supplemental information

Document S1. Supplemental Methods, Supplemental Figures 1–3, and Supplemental Tables 1 and 2

Supplemental Data 1. *Xa7* from 3000 rice genomes

Document S2. Article plus supplemental information
