# Supplementary material for: The Xa7 resistance gene guards the rice susceptibility gene SWEET14 against exploitation by the bacterial blight pathogen
Source: Plant Commun. 2021 Jan 19;2(3):100164. doi: 10.1016/j.xplc.2021.100164 (PMC8132128; doi:10.1016/j.xplc.2021.100164)
Supplement: Document S1. Supplemental Methods, Supplemental Figures 1–3, and Supplemental Tables 1 and 2 [file mmc1.pdf]

Plant Communications, Volume *2*

Supplemental information

The *Xa7* resistance gene guards the rice susceptibility gene *SWEET14* against exploitation by the bacterial blight pathogen

Dangping Luo, Jose C. Huguet-Tapia, R. Taylor Raborn, Frank F. White, Volker P. Brendel, and Bing Yang

The *Xa7* Resistance Gene Guards the Susceptibility Gene *SWEET14* of Rice Against Exploitation by Bacterial Blight Pathogen

Dangping Luo[1], Jose C. Huguet-Tapia[2], Taylor R. Raborn[3], Frank F. White[2], Volker P. Brendel[3], Bing Yang[1,4,*]

[1] Division of Plant Sciences, Bond Life Sciences Center, University of Missouri, Columbia, MO 65211, USA

[2] Department of Plant Pathology, University of Florida, Gainesville, FL 32611, USA

[3] Department of Biology, Department of Computer Science, Indiana University, Bloomington, IN 47405, USA

[4] Donald Danforth Plant Science Center, St. Louis, MO 63132, USA

[*] Correspondence: Bing Yang (yangbi@missouri.edu)

Files:

Supplemental Table 1. Molecular markers used in this study

Supplemental Table 2. Oligonucleotides used in this study

Supplemental Information 1. The cDNA sequence of *Xa7*

Supplemental Information 2. gBlocks of *Xa7* homologous genes

Supplemental File 1. Program for genome assembly

Supplemental Figure 1. Large chromosomal fragment deletion of *Xa7* locus

Supplemental Figure 2. Screenshot of RAMPAGE analysis displayed with the Integrative Genomics Viewer (Thorvaldsdóttir, Robinson et al. 2013)

Supplemental Figure 3. *Xa7* and homologs in diverse grass species

Supplemental Table 1. Molecular markers used in this study.

Marker	Primer	Size in IRBB7 (bp)	Size in Nipponbare (bp)
RM7243	RM7243F	150	158
	RM7243R		
RM20571	RM20571F	123	117
	RM20571R		
RM5509	RM5509F	229	255
	RM5509R		
5610	5610F	261	231
	5610R		
M5	M5-F	343	1219
	M5-R		
M5-3k	M5-3kF	143	122
	M5-3kR		
M5-5k	M5-5kF	107	104
	M5-5kR		
M5-48k	M5-48kF	152	144
	M5-48kR		
M5-72k	M5-72kF	127	136
	M5-72kR		
RM20593	RM20593F	314	316
	RM20593R		
RM3723	RM3723F	130	138
	RM3723R		

Sequences of each markers in IRBB7 and Nipponbare

RM7243

IRBB7
AAGATGGCGTGCGTACGTACGTACGTGCGGCGCGGGCGTACAGGCGTACAGGGCGC
CGCGCGACGCATGGATGGATGGATCGATGGATCGATGGTCCCGGGCGGCACAGGCA
GGGGCTCCCCCGACCGGGCAACCCGTGAAGAACTTCGT

Nipponbare
AAGATGGCGTGCGTACGTACGTACGTGCGGCGCGGGCGTACAGGCGTACAGGGCGC
CGCGCGACGCATGGATGGATGGATGGATGGATCGATGGATCGATGGTCCCGGGCGG
CACAGGCAGGGGCTCCCCCGACCGGGCAACCCGTGAAGAACTTCGT

RM20571

IRBB7
GAGAGGTGGAGAGATGGATGTGGAGGGTGGTGGTGGTGGTGGTGGTGGTGGCG
CGCCGCCCAGGGGGAGGAATTCTTGGGTAATCAATTAAGCTTTGGTTCTTGGTTTGG
TTCTTTGGTC

Nipponbare
GAGAGGTGGAGAGATGGATGTGGAGGGTGGTGGTGGTGGTGGTGGTGGCGCGCCGC
CCAGGGGGAGGAATTCTTGGGTAATCAATTAAGCTTTGGTTCTTGGTTTGGTTCTTTG
GTC

RM5509

IRBB7
GATGATCCATGCTTTGGCCGGCCCACGGATTTCGTTTTTTTTTTTTTGTGGCAATTTA
ACATCCTCATATGCTAGATTTTTCTTAGGCCAATATGGTCTCTCTCTCTCTCTCTCTCT
CTCTCTCTCTCTCTCTGAGCAATGAAAAAGATTTGATAGGGATATTTGATGAAAGCT
TAACTGATTACTATTAAATTTATAATGATCATTGTGCGTCTTCTTTCTGCTGGAA

Nipponbare
GATGATCCATGCTTTGGCCGGCCCACGGATTTCGTTTTTTTTTGTGTGGCAATTTAAC
ATCCTCATATGCTAGATTTTTCTTAGGCCAATATGGTCTCTCTCTCTCTCTCTCTCTCT
CTCTCTCTCTCTCTCTCTCTCTCTCTCTCTCTCTCTCTCTTTGAGCAATGAAAAAGATT
TGATGGGGATATTTGATGAAAGCTTAACTGATTACTATTAAATTTATAATGATCATT
GTGCGTCTTCTTTCTGCTGGAA

5610

IRBB7
AAGCTCGTCACCTCACCCGTCACTTCACCAGCCTGTAGCCTCTCTCTCTCTCTCCGTG
ATTTTGGTTAATTTGGAGGCCGTGCCCTGCCGGATGGATGGGCATTGATGGACGCGC
CGGCCGTGGAGACGTCGCGTCCACCGACCCACCGCGCCCCTGCGCGCAACGCAACA
AACGCGCGCAACCGCGATCGATAGCCGGCATCTCCTCCTTCCACGTACCAGCCGAG
GAGCCGAGCACTGCAGATATGCTGCTACTGTGCA

Nipponbare
AAGCTCGTCACCTCACCCGTCACTTCACCAGCCTGTAGCCTCTCTCTCTCTCTCTCTC
TCCGTGATTTTGGTTAATTTGGAGGCCGTGCCCTGCCGGATGGATGGGCATTGATGG
ACGCGCCGGCCATGGAGACGTCGCGTCCACCGACCCACCGCGATCGATAGCCGGCA
TCTCCTCCTTCCACGTACCAGCCGAGGAGCCGAGCACTGCAGATATGCTGCTACTGT
GCA

M5

IRBB7
CGATCTTACTGGCTCTGCAACTCTGTATTGCATGCTAAATCCGTTATGATTTACTACT
AATGATGGATGTACTGATACATAGTACTGATCGATGAGCTAGCTAGGGTTTTGGATG
GGTCGGAAGGTGAGAAAGAGGAGGAAAGAAGAGAGAATTGAAGATGATTAGAAGA
AATCTAATCAATTTCAGAGAATTACTGTATTCATCTTTCTGTGTATATTCGTCTGGGT
AGTGCTGATTGTGCCATTATAATTGACGGGGTCGTACTTCGTACGGACGAATCGACA
CAGACATGCATGATTGTCTTTTAGTACTTAGAATTAGTAGCAAAGTCGGTGGGACAA
C

Nipponbare
CGATCTTACTGGCTCTGCAACTCTGTATTGCATGCTAAATCCGTTATGATTTACTACT
AATGATGGATGTACTGATACATAGTACTGATCGATGAGCTAGCTAGGGTTTTGGATG
GGTCGGAAGGTGAGAAAGAGGAGGAAAGAAGAGAGAATCTATTATATTATTAAAG
GAATAGAAAAAGAAGCCTCCACGTTCGCTCTCACGGCCTAGAAATTCTCACATTAAT
CGGAGAAAAGAAAAAGCAGAGTCCATATAGAAATACAATTTAGAAATAGCTGAAAT
TCGGAATTATAAAATAAGGAATATTAGAAGAGGAGACTAGAGTCCATATGGAAATA
CAATTTAGAAATAGTTGAAATTCAGAATTAAAAAATAAGAAATATTAGAAGAGGAG
ACTAGAGTCCATATAGAAATATAATTAGGAAATAACTGAAATTCGGAATTAAAAAT
AAGGAATATTAGAAGTAGAGTATAGAGTCCATATAAAAATATAATTAGGAAATAAC
TGAAATTAGGAATTAATAATAAGGAATATTAGAGATAGAGTATAGAGTCCATATAA
AAATACAATTAGTAAATAACTGTAATTCGGAATTAAAAATAAGGAATATTAGAGGT
AGAGTATAGAGTCCATATAGAAATACAATTAGAAAATAACTGTAATTCGGAATTAA
AAATAAGGAATATTAGAGGTAGAGTATAGAGTCCATATAAAAATACAATTAGGAAA
TAACTGAAATTCGGAATTAAAAATAAGGAATATTAGAGGTAGAGTATAGAGTCCAT
ATAGAAATACAATTAGAAAATAATAAAAATTCGGAATTAAAAATTTGATATTAAAA
ATAATTAATAACTAACACGTATATATAATACAATATAAATATTACACATTAGTAGTT
TTACAAAATTTAAAATTATATTGTCATTTTAATAAATTTGAATAATACATTGAGAAA
ACATATATGCTATTACATGAGAGAAAATATAATGATGCTAGCCGCGCAATATGCAC
GGGCCACTATGCTAGTTGAAGATGATTAGAAGAAATCTAATCAATTTCAGAGCATTA
CTGTATTCATCTTTCAGTGTATATTCGTCTGGGTAGTGCTGATTGTGCCATTATAATT
GACGGGGTCGTACTTCGTACGGACGAATCGACACAGACATGCATGATTGTCTTTTAG
TACTTAGAATTAGTAGCAAAGTCGGTGGGACAAC

M5-3k

IRBB7
CATATGTAGCAAGTATGCATCCAGCGAAACCCCATGGATTAATTAATTTGGCGGTAG
ATCTCATCATAACAGAGAGTACGTACGTAGTAGTACTGTAGTACACTTGGATATATA
CAAATTAAGCCAACACATTAGTCTTCAGA

Nipponbare
CATATGTAGCAAGTATGCATCCAGCGAAACCCCTAGATCTCATCATAACAGAGAGT
ACGTACGTAGTAGTACTGTAGTACACTTGGATATATACAAATTAAGCCAACACATTA
GTCTTCAGA

M5-5k

IRBB7
TGTAGAGATTGTGACGAGGAGCAGTGCAAGTTTCAGGTAGTGATGCATCAGGCGTTT
GCCCTCTTTCCTTGCAATGCTACTCGCTGATGATCCATTCTGCAACATCA

Nipponbare
TGTAGAGATTGTGACGAGGAGCAATGCAAATTTCAGGTAGTGATGCGTCAGGCGCT
TGCCCTCTTTCCTTGCAGTGCTACTCGCTGATCCATTCTGCAACATCA

M5-48k

IRBB7

CTGTGTGAAAGTTCAGACGGATGGCGATTTCGCAAAGATGTACTCCATTTGAGATCT
TTATGTGTATGTATGTATACTTGTCAGGTCTGATGGAGGTCCAAAGAAATGGCAGTC
TACTGATGCTGGAATGCAGTCTCATGTGCTCAAAGCAG

Nipponbare
CTGTGTGAAAGTTCAGACGGATGGCGATTTTGCAAAGATGTACTCCATTTGATATCT
TTGTGTGTATACTTGCCAGGTCTGATGGAGGTCCAAAGAAATGGCAGTCTACTGATG
CTGGAATACAGTCTCATGTGCTCAAAGCAG

M5-72k

IRBB7
TGGAAAGGAAGCTCCTCGACGGCGGCGTGGGCGGCGCCGCGGCGGCGGTCACCATC
TACGGCTGCCCGGTGATGGTCGACGTCGATCTCAACAAGTGCGAGCCATGGGACCTT
CCTGGTAAGACCTA

Nipponbare
TGGAAAGGAAGCTCCTCGACGGCGGCGTGGGCGGCGCCGCGGCGGCGGCGGCGGC
GGTCACCATCTACGGCTGCCCGGTGATGGTCGACGTCGATCTCAACAAGTGCGAGCC
ATGGGACCTTCCTGGTAAGACCTA

RM20593

IRBB7
AAGGTACACTTGCTCTGACGGTAGCAAAAGTTGATCATGCCCTACTTTAGCTGTAAC
ATGAGACATGTCAGCATATGCTTACATGCATGAATCAGCAGGGCTACAGTTTTATTA
TTTGCTTTGACAGCTTTCTCTCAACAATCATTCTCATTATATTTTTTATCCTAGGCTCC
TTAGGAACATAGTAATTTTACAGGATTTTTGCTGAAATCGATCAGTTCAGTTCCCGC
AAAATTCCACTGAAAATTAATCCTCTACTCCAAAGGCCTCTCTCTCTCTCTCTCTCTC
TCACGTAGGATTTGCCACTGAGGTCT

Nipponbare
AAGGTACACTTGCTCTGACGGTAGCAAAAGTTGATCATGCCCTACTTTAGCTGTAAC
ATGAGACATGTCAGCATATGCTTACATGCATGAATCAGCAGGGCTACAGTTTTATTA
TTTGCTTTGACAGCTTTCTCTCAACAATCATTCTCATTATATTTTTTATCCTAGGCTCC
TTAGGAACATAGTAATTTTACAGGATTTTTGCTGAAATCGATCAGTTCAGTTCCCGC
AAAATTCCACTGAAAATTAATCCTCTACTCCAAAGGCCTCTCTCTCTCTCTCTCTCTC
TCTCACGTAGGATTTGCCACTGAGGTCT

RM3723F

IRBB7
TAGACATGGGTCCCTCACAGATGGAACCCATTCTCATAGGCTCACATGTCAGCGGAC
ACATATTCCTCTAACTTAGAGGAGCCTCCCCCTAATGAAAGTGTAATATAGGATCAG
AAGCAAGACTTGTGGA

Nipponbare
TAGACATGGGTCCCTCACAGATGGAACCCATTCTCATAGGCTCACATGTCAGCGGAC
ACATATTCACATATTCCTCTAACTTAGAGGAGCCTCCCCCTAATGAAAGTGTAATAT
AGGATCAGAAGCAAGACTTGTGGA

Supplemental Table 2. Oligonucleotides used in this study

Oligos	Sequence (5′ TO 3′)	Usage
RM7243F	ACGAAGTTCTTCACGGGTTG	Mapping
RM7243R	AAGATGGCGTGCGTACGTAC	
RM20571F	GAGAGGTGGAGAGATGGATGTGG	
RM20571R	GACCAAAGAACCAAACCAAGAACC	
RM5509F	GATGATCCATGCTTTGGCC	
RM5509R	TTCCAGCAGAAAGAAGACGC	
5610F	AAGCTCGTCACCTCACCCGTCAC	
5610R	TGCACAGTAGCAGCATATCTGCAG	
M5-F	GTTGTCCCACCGACTTTGCTA	
M5-R	CGATCTTACTGGCTCTGCAACTCT	
M5-3kF	CATATGTAGCAAGTATGCATCC	
M5-3kR	TCTGAAGACTAATGTGTTGGCT	
M5-5kF	TGTAGAGATTGTGACGAG	
M5-5kR	TGATCCATTCTGCAAC	
M5-48kF	CTGTGTGAAAGTTCAGACGG	
M5-48kR	CTGCTTTGAGCACATGAGAC	
M5-72kF	TGGAAAGGAAGCTCCTCGAC	
M5-72kR	TAGGTCTTACCAGGAAGGTC	
RM20593F	AAGGTACACTTGCTCTGACGGTAGC	
RM20593R	AGACCTCAGTGGCAAATCCTACG	
RM3723F	TAGACATGGGTCCCTCACAGATG	
RM3723R	TCCACAAGTCTTGCTTCTGATCC	
3628F	TGTCCGGTGTCTCTAGCTTC	
3628R	ACCAAGCTATACCAAGTACT	
5760-14kF	CTCAACACTCCTCTTCACAG	
5760-14kR	AGTGAGAGAAGCGTAGAGAC	
RM53266F	AGCCTTCCTAACCAATCTGG	
RM53266R	CCATCATTTCGGCTCTTCAC	
3770F	GTCACCAGTCACCACCGATC	
3770R	CGTGATAACCGCGAGACATA	
gRNA1-F	GCAGGTACGTATACCAGTTGTCCG	For the 1st guide RNA to delete the 53 kb of *Xa7* locus
gRNA1-R	AAACCGGACAACTGGTATACGTAC	
gRNA2-F	GCAGGTTTGTCCAATGAGTTGCTG	For the 2nd guide RNA to delete the 53 kb of *Xa7* locus
gRNA2-R	AAACCAGCAACTCATTGGACAAAC	
gRNA3-F	TGTTGGATGATCAGCGGCCGCCA	For guide RNA at the 1st site of *Xa7*
gRNA3-R	AAACTGGCGGCCGCTGATCATCC	
gRNA4-F	GTGTGAGGAAGACGCCGGAGTTGA	For guide RNA at the 2nd site of *Xa7*
gRNA4-R	AAACTCAACTCCGGCGTCTTCCTC	
Xa7aRT-F	CGTATGCCCGTTGCAGTTGCAG	RT-PCR for *Xa7* expression
Xa7aRT-R	CGGAGTTGACGGTCAGCAGTCG	
Xa27L-F4,	ATAGGCCGGTATCCCAGCTCT	For genotyping *Xa7* CRISPR plant
112.5R	ACTGGAGCAGAGGAATGTGC	
EP53-F	TGTAGAAACCATGTCTCATGC	For genotyping large deletion
BTB-R1	ACTACCACACCAGCCAGCAAG	
EP53R	CTTGGATGGAGCTCCAGATG	With EP53-F as internal primers for zygosity of large deletion plants
Pro2.7k HindF	TATGACCATGATTACGCCAAGCTTC TCATCCCAACCGTTCTG	Amplify 2.7 kb *Xa7* promoter
Xa7aATG-R	AGAAATTTACCCTCAGATCTACCAT GGATGATGGATCCCCCAG	
DelEBEF3	ATACGAACGAAGGCTTTGAAGC	Delete 20 bp for mutant EBE

DelEBER3	TTCAAAGCCTTCGTTCGTATGGGTTATATATTGGTTTAGCAG	

Supplemental Information 1. The cDNA sequence of *Xa7*.

CCCCTTCCCAACCACAGCCAGCGGTTTCCAAACTCCACGCCTCGCTCAACCTGGGGGATCCATCATCCATGGCGGCC
GCTGATCATCCTGATCGTATGCCCGTTGCAGTTGCAGGCTTGCGCCACCATTACGCCTTCCcTGCAAACCTTCGCCC
CGCCGCTCGACTGCTGACCGTCAACTCCGGCGTCTTCCTCATCTCCACCGCCGGGGCCATCGTCCTCGTCCACACCG
CCGGTAACCCACCCGCCATCGACAACGATCCAGCCTACGCCTTGGTCGCATTCGTGCTCTTCCTCCTCGGAATCTGG
CTCATGTCTATTGCCCTCGTCGCCGACCAGTTCCcGCGCGCCGCTGGGGTCGCCGTGGCCATTGCCAGGGCGCTGCA
GGATTACCTCATCGGTGGCAATTAACTAGAAGCTTCGACCATGGCTCTGCACATTCcTCTGCTCCAGTTGTTCCCGG
CTTCCCGTACGTGTGCCTGATGATTGTCTTTCTCTGTTTATTTGGCTAGTATTTTAGGCTTGGAAGTTGAAAAACTG
TAAATCTGCTTCTTTTTCCCCTCTGTACTACTACTAGACTTTCTTTTTTAaGCTGACGTCATACACACacCCCAGAT
TCATAACGTGTCGTATAGTAAATGTATTCGAGGCTTGTAATAAAaaGGCACCCGTAGGTGTATGCTCTGTTCAGTCT
GATGTTCTAAATAATCAAGATACTATTAGTCGT

Supplemental Information 2. gBlocks of *Xa7* homologous genes.

gBlock cloned into pBY02 at EcoRI-HindIII through Gibson cloning. Sequences shaded in yellow are overlapping regions to facilitate the insertion of gBlocks in pBY02 through Gibson cloning. The start and stop codons are in red. Sequences for EcoRI and HindIII are underlined. The Kozac sequence for optimal expression of gene under 35S promoter is in bold.

Do_Xa7 gBlock
GAGAACACGGGGGACTCTAGGAATTC**GATCTACC**ATGGCGGACCGGCCGGCAGCAATCCATCAGGGCCTGGCGCCGG
TGGGCTTTGTGGTCCGGCAACAGCGAGCGATCGCCCCTCGTCGCGCCCGACTCCTGATGATCAATTCCGGCACCCTC
CTGATCTCGGCCGCCTGGTCCGTCATCATCATCCACAGCACTTCATCAGGCACTACAGCCGGTGGTGGTCGGGCTTT
CGCCCTCGTCACATTCCTCCTCTTCCTTCTCGGCGTGTCGCTGGTGATGGTGGCGCTTGTCGCTTACAGGTTCCGGC
GAGCGGCAGCGGTCGGCGTGGCCATTGCGAGGGCGCTCCGCCGCTACTTGCTCGGTCTTGGGTGGTAAGCTTCTAGA
CTAGTGAGCTCGAAT

Si_Xa7 gBlock
GAGAACACGGGGGACTCTAGGAATTC**GATCTACC**ATGGCGGGCCGGCCAGCAGAACTCCATCTCCAGCGAGTGATCG
CAGGCCGCCGTGCGCGGCTCCTGGTCAACGCCGGCGCCCTGCTCATCTCGGCCGCCGGGTCCGTCATCATCCACGCC
GCTGCCACGCCCTCTGACGCTTCCTCTGGCCCGGCGCGCCCGCTCATCGCGTTTTGCATCTTCGTCCTCGGCGTGTC
GCTGGTGATGTCGGCGCTCGTCGCCGACCGGTTCCCAAGAGCGGCAAGGGCCGGCGTGGCCATTGCGAGGGCACTCC
AACGCTACGTGTTCGGTCTTGTTGGGTGGTAAGCTTCTAGACTAGTGAGCTCGAAT

Ph_Xa7 gBlock. (codon optimized for *N. benthamiana* due to high GC of the original sequence)
GAGAACACGGGGGACTCTAGGAATTC**GATCTACC**ATGGCCGGTCTCCAACCAGCAGCCATCCACCTTCAACGTAGAC
AACAGGCTATTGCTGGTAGGCGAGTACGTCTCTTAGTAAACGCAGGAGCACTGTTGGTATCCGCAGGAGGCAGTGTC
GTTATCATTCACGCAGCTACTCCTTCAGACGACGCCGCAGGAGGGCCCGCCTGCTCTCTGGTGGCCTTTTCTGTCTT
TCTGCTGGGAGTCTCTTTAGTCACGCTGGCCTTAGCTGCTGATAGATTCCCACGTGCAGCAAGGGTTGGGGCCGCCG
TAGCAACAGCTACTAATAGGTATTTGTTTGGACTCGGCTGGTAATAAGCTTCTAGACTAGTGAGCTCGAAT
gBlock cloned into pBY02 at EcoRI-HindIII through Gibson cloning.

Sb_Xa7 gBlock (codon optimized for *N. benthamiana* due to high GC of the original sequence)
GAGAACACGGGGGACTCTAGGAATTC**GATCTACC**ATGTCTCGACCCGCCGAGATGCGTAACCCCCGTCACGCAGACG
ATGGCGATTTGCAGTTAGCTTGGATTGGTAGAAGGCGAAGGGGCCTTCAACAACAACTCCTGTTGGATTCCGGAGGC

AGGGCCTGCATGTTGTTAGGGGCTCTGGTCCTCACTTGGGATCAGCTCGCCCTCGCAAGTTCTTCCAGCCCCGAGCA
TGTCCTGCTGGCTGCATTTGTTCTTTGGTTACTGGGGGCCGCCCTCGTGATGCTTAGTCTGGTAAGCCGAAGGTTTC
CAAGACTGGCATCCGCCGGTGCAGCCCTGGTAATGGCACTTAGGAATTATTTGTTAGGGGGTGGTGGGCTCTAAGCT
TCTAGACTAGTGAGCTCGAAT

Op_Xa7 gBlock
GAGAACACGGGGGACTCTAGGAATTC**GATCTACCATG**GTGAACCGGACAGCCGCTCATCCGCAGATGCCTGTTCAGG
CGCTGCGCCGCAATCGCATCGCGCAGGTAAACAGGGTGCCCGCCGCTCGCCGTGCTCGAGCCCGACTGCTGATCGTC
AACTCCGGCGTTTTCCTCATCTCCACCTCCGGAGCCATCGTCGTCGTCCACACCGCTGGAAACCCCTTCGCCCTCGT
CGCATTCGTGCTCTTCCTACTTGGCATCTGGCTCGTGCTTCTTGCCCTCGTCGCCGACAAGTTCCCGCGAGCCGCCA
GGGTCGCCGTCGCCATTGCCAGTGCACTGCAGGATCACCTCATAGGCGGCAACTAGTAAGCTTCTAGACTAGTGAGC
TCGAAT

Ol_Xa7 gBlock
GAGAACACGGGGGACTCTAGGAATTC**GATCTACCATG**GCGAATCGGACAGCTGCTCATCCGCGGATGCCCGTACAGG
GGCTGCGCCGCAATCAAATCGCGCGGGGGCCCGCCGCTCGCCGTTCTCGACTGCTGATCGTCAACACCGGCGTTTTC
CTCATCTCCACCTCCGGGGCCATCGTCGTCGTCCACACCGCTGGAAACCCCTCCTCCGCCATCGACGACGGTCCGTC
CTCCGCCCTCGTCGCATTCGTGCTCTTCCTACTTGGCATCTGGCTCGTGCTTCTTGCCCTCGTCGCCGACAAGTTCC
CGCGAGCCGCCAGGGTCGCCGTCGCCATTGCTAGTGCACTGCAGGATCACCTCATAGGCGGCAACTAGTAAGCTTCT
AGACTAGTGAGCTCGAAT

>Ol_XA7 KN541332.1_FGP003 (*Oryza longistaminata*)
MANRTAAHPRMPVQGLRRNQIARGPAARRSRLLIVNTGVFLISTSGAIVVVHTAGNPSSAIDDG
PSSALVAFVLFLLGIWLVLLALVADKFPRAARVAVAIASALQDHLIGGN

>Op_XA7 OPUNC06G22220 (*Oryza punctata*)
MVNRTAAHPQMPVQALRRNRIAQVNRVPAARRARARLLIVNSGVFLISTSGAIVVVHTAGNPFA
LVAFVLFLLGIWLVLLALVADKFPRAARVAVAIASALQDHLIGGN

>Do_XA7 BAE44_0010678 (*Dichanthelium oligosanthes*) (NCBI accession: OEL28303.1)
MADRPAAIHQGLAPVGFVVRQQRAIAPRRARLLMINSGTLLISAAWSVIIIHSTSSGTTAGGGR
AFALVTFLLFLLGVSLVMVALVAYRFRRAAAVGVAIARALRRYLLGLGW

>Sl_XA7 LOC105914264 (*Setaria italica*) (NCBI accession: XP_012700797.1)
MAGRPAELHLQRVIAGRRARLLVNAGALLISAAGSVIIHAAATPSDASSGPARPLIAFCIFVLG
VSLVMSALVADRFPRAARAGVAIARALQRYVFGLVGW

>Ph_XA7 LOC112891075 (*Panicum hallii*) (NCBI accession: XP_025813762.1)
MAGLQPAAIHLQRRQQAIAGRRVRLLVNAGALLVSAGGSVVIIHAATPSDDAAGGPACSLVAFS
VFLLGVSLVTLALAADRFPRAARVGAAVATATNRYLFGLGW

>Sb_XA7 SORBI_3K004400 (*Sorghum bicolor*) (NCBI accession: OQU75609.1)
MSRPAEMRNPRHADDGDLQLAWIGRRRRGLQQQLLLDSGGRACMLLGALVLTWDQLALASSSSP
EHVLLAAFVLWLLGAALVMLSLVSRRFPRLASAGAALVMALRNYLLGGGGL

Supplemental File 1. Program for genome assembly.

```
###The script uses illumina reads dowloaded form the 3K rice genomes project
#Raw sequence data is also available from the http://gigadb.org/dataset/200001 - SRA at PRJEB6180
#deposited the reads in a new folder rice3k
## use the Xa7 region as reference and create an index using bowtie2
bowtie2-build complete_Xa7.fasta cXa7

##create an accesion list from the reads_dictionary that contains the sample accesion and the run id
## sample_accession  (1st collumn) run_accession (2dn collum) in reads_dictionary
for x in `cut -f 1 reads_dictionary`
do
grep $x reads_dictionary | sed 's/$//g' | cut -f 2 > copy_list
####
	for i in `cat copy_list`
	do
	cp "/rice3k/"$i"_1.fastq.gz" ./
	cp "/rice3k/"$i"_2.fastq.gz"  ./
	gunzip *.fastq.gz
	done
###
##generate temporary files to map
cat *_1.fastq > $x"_1.fq"
cat *_2.fastq > $x"_2.fq"
rm *_1.fastq
rm *_2.fastq
rm *.fastq.gz
###

bowtie2 -x cXa7 -1 $x"_1.fq" -2 $x"_2.fq" -S $x"_results.sam" -p 8
rm *.fq

samtools view -bS -o $x"_results.bam" $x"_results.sam"
rm $x"_results.sam"

samtools view -b -F 4 $x"_results.bam" > $x"_mapped.bam"
rm *_results.bam

samtools sort $x"_mapped.bam" -o $x"_results_mapped.sorted.bam"

rm $x"_mapped.bam"

samtools index $x"_results_mapped.sorted.bam"
####
done
```

###Generate a consensus Xa7 for each mapped rice3k
sam_list is the list of bam files generate above

```
for x in `cat sam_list`
do
samtools mpileup -uf complete_Xa7.fasta $x"_results_mapped.sorted.bam"  | bcftools call -c |
vcfutils.pl vcf2fq > $x"_cns.fq"
done
```

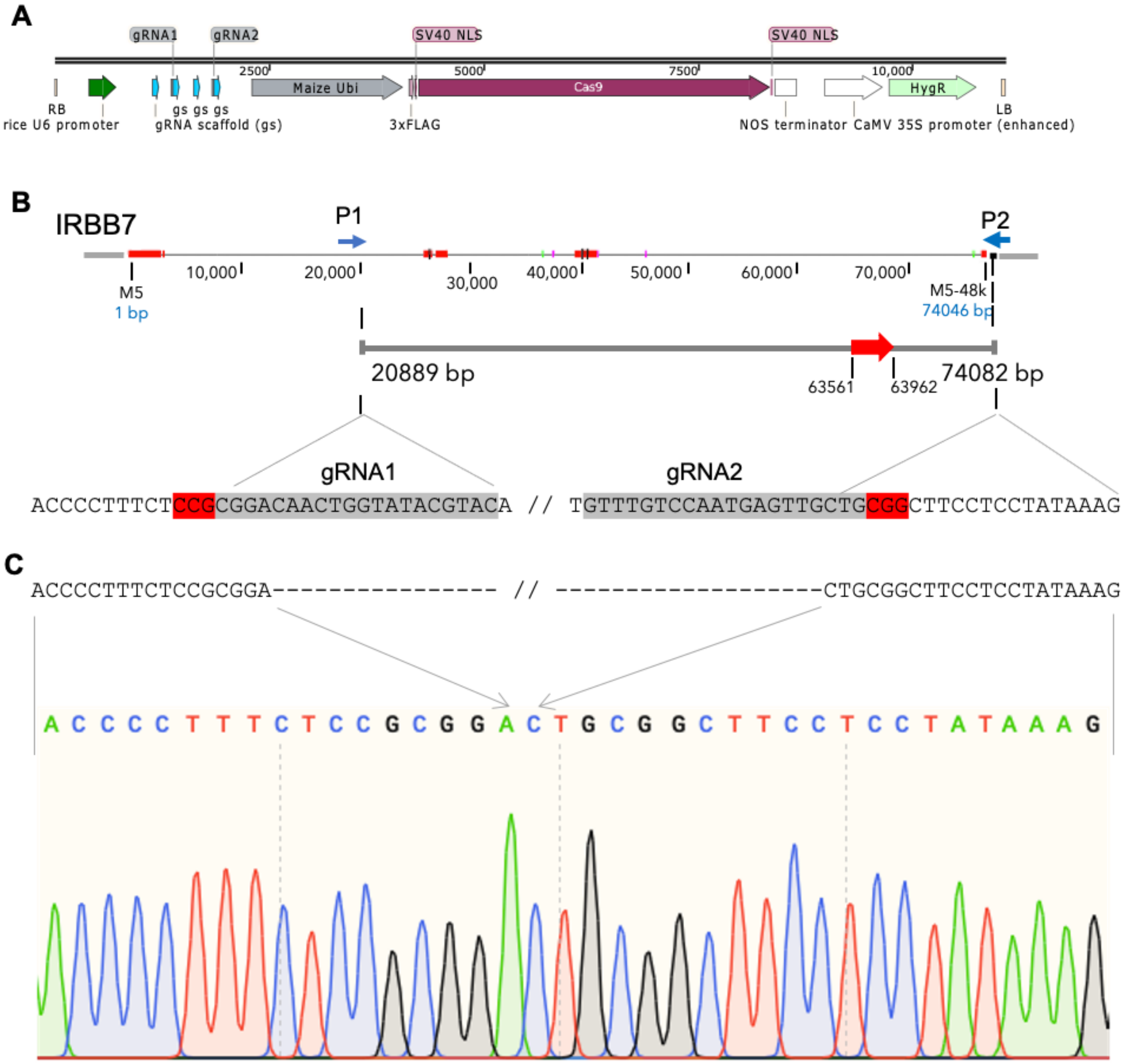

Supplemental Figure 1. Large chromosomal fragment deletion of *Xa7* locus**.**

(A) Schematic map of the CRISPR/Cas9 construct used for genome editing of *Xa7* locus. Guide RNA guides are under rice U6 promoters and Cas9 under maize ubiquitin 1 gene promoter. Hygromycin resistance gene is under the CaMV 35S promoter.
(B) *Xa7* locus delimited by two markers with guide RNA target sites. P1 and P2 are two deletion-specific primers to screen and identify the CRISPR-induced large deletion lines. Guide RNA target sites are in shaded with adjacent Cas9 PAM (protospacer adjacent motif) shaded in red.
(C) Sequencing chromatogram of PCR-amplicon from the T0 nb7-1 line. PCR-amplicon with two primers (P1 and P2) and genomic DNA of nb7-1 was subjected to Sanger sequencing.

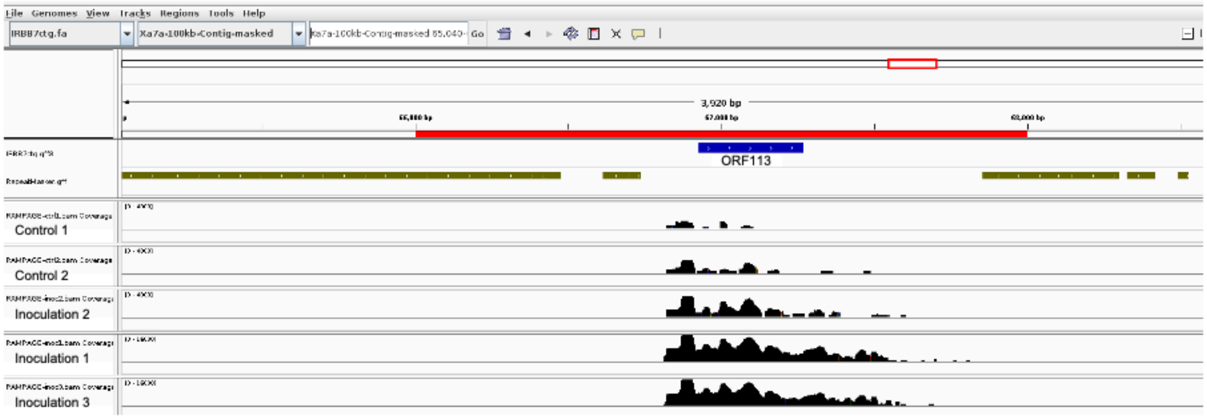

Supplemental Figure 2. Screenshot of RAMPAGE analysis displayed with the Integrative Genomics Viewer (Thorvaldsdóttir, Robinson et al. 2013).

RAMPAGE reads from two RNA samples derived from IRBB7 leaves inoculated with MX53 (Control 1 and 2) and three RNA samples (Inoculation 1, 2, and 3) derived from IRBB7 leaves inoculated with PXO86 were used for analysis. The tracks show read coverage per position. Note that Control 1 and 2 and Inoculation 2 coverage is shown on a scale 0-4000, compared to 0-16000 for Inoculation 1 and 3, in order to accommodate different library sizes (read pairs aligned are 7,021,714; 8,098,348; and 6,507,082 for Control 1 and 2 and Inoculation 2, respectively; compared to 32,296,182 and 37,344,426 for Inoculation1 and 3, respectively); Alignment and analysis were done with GoRAMPAGE (https://github.com/BrendelGroup/GoRAMPAGE). Counts are displayed on a log scale. For example, site 66,859 has coverage 9 and 31 for Control, compared to 9144, 217, and 6100 for Inoculated.

Thorvaldsdóttir, H., J. T. Robinson and J. P. Mesirov (2013). "Integrative Genomics Viewer (IGV): high-performance genomics data visualization and exploration." Brief Bioinform **14**(2): 178-192.

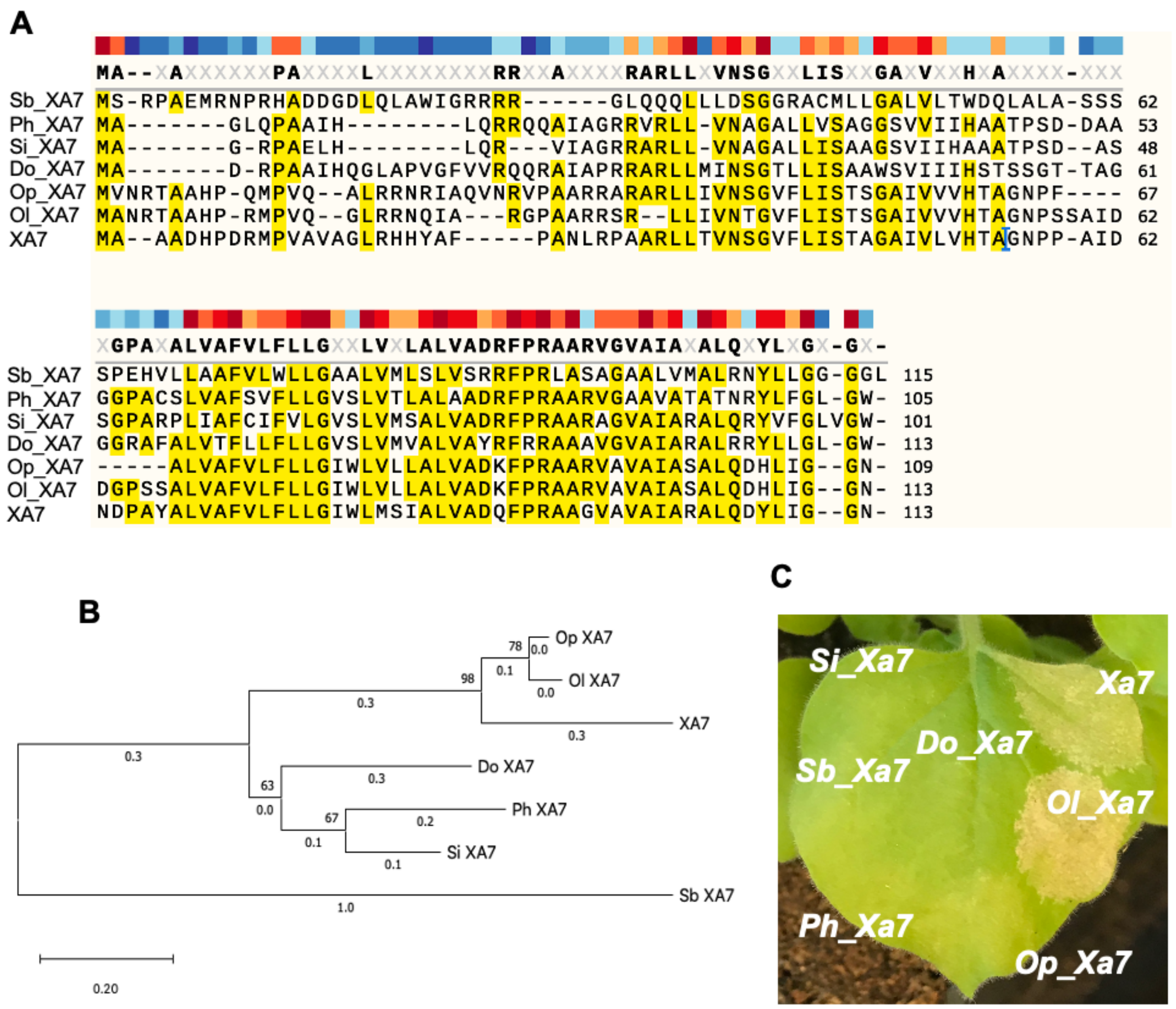

Supplemental Figure 3. *Xa7* and homologs in diverse grass species.

(A) Amino acid alignment of seven XA7 homologs generated by ClustalX. Sequences are from *Do_Xa7*, *Dichanthelium oligosanthes*; *Si_Xa7*, *Setaria italica*; *Ph_Xa7*, *Panicum hallii*; *Sb_Xa7*, *Sorghum bicolor*; *Op_Xa7*, *Oryza punctata*; *Ol_Xa7*, *Oryza longistaminata*.
(B) Phylogenetic tree of seven XA7 homologs. The unrooted phylogenetic tree of 7 proteins was generated by using the Maximum Likelihood method and JTT matrix-based model. The tree with the highest log likelihood (-1290.17) is shown. The tree is drawn to scale, with branch lengths measured in the number of substitutions per site (next to the branches). The analyses were conducted in MEGA X (http://www.megasoftware.net).
(C) Cell death phenotypes caused by overexpression of *Xa7* and its homologs in *N. benthamiana* through agroinfiltration. Leaf image was taken three days post infiltration.
